# Association between *MTHFR* (677C>T and 1298A>C) polymorphisms and psychiatric disorder: A meta-analysis

**DOI:** 10.1371/journal.pone.0271170

**Published:** 2022-07-14

**Authors:** Xinyao Meng, Ji-long Zheng, Mao-ling Sun, Hai-yun Lai, Bao-jie Wang, Jun Yao, Hongbo Wang

**Affiliations:** 1 School of Basic Medicine, Shenyang Medical College, Shenyang, P.R. China; 2 Department of Forensic Medicine, China Criminal Police College, Shenyang, P.R. China; 3 School of Forensic Medicine, China Medical University, Shenyang, P.R. China; National Institutes of Health, UNITED STATES

## Abstract

Recent studies showed that genetic polymorphism of 5,10-methylenetetrahydrofolate reductase (MTHFR) is related to attention-deficit hyperactivity disorder (ADHD), bipolar disorder (BD) and schizophrenia (SCZ). However, no consistent conclusion has been determined. This meta-analysis aims to interrogate the relationship between *MTHFR* gene polymorphisms (677C>T and 1298A>C) and the occurrence of ADHD, BD and SCZ. We retrieved case-control studies that met the inclusion criteria from the PubMed database. Associations between *MTHFR* polymorphisms (677C>T and 1298A>C) and ADHD, BD and SCZ were measured by means of odds ratios (ORs) using a random effects model and 95% confidence intervals (CIs). Additionally, sensitivity analysis and publication bias were performed. After inclusion criteria were met, a total of five studies with ADHD including 434 cases and 670 controls, 18 studies with BD including 4167 cases and 5901 controls and 44 studies with SCZ including 16,098 cases and 19913 controls were finally included in our meta-analysis. Overall, our meta-analytical results provided evidence that the *MTHFR* 677C>T was associated with occurrence of BD and SCZ, while the 1298A>C polymorphism was related to ADHD and BD, and additionally the sensitivity analysis indicated these results were stable and reliable. This may provide useful information for relevant studies on the etiology of psychiatric disorders.

## Introduction

Folic acid, a member of the vitamin B complex, in considered to be strongly associated with the function and development of the central nervous system, which plays an important role in cellular processes including nucleotide synthesis and methylation [1]. The enzyme 5,10-methylenetetrahydrofolate reductase (MTHFR) functions in the pathway that converts folate into metabolites that may be used for cellular processes including methylation of gene promoter enhancers and protein, RNA, DNA, amino acid and phospholipid synthesis. Specifically, this enzyme converts 5,10-methylenetetrahydrofolate to 5-methyltetrahydrofolate, which is required for the multistep process that converts the amino acid homocysteine to methionine. Methionine is used to synthesize proteins and other important compounds [2]. The *MTHFR* gene is located at 1p36.22 [3]. Genetic variation in this gene influences susceptibility to occlusive vascular disease, neural tube defects, colon cancer and acute leukemia, and mutations in this gene are associated with MTHFR deficiency. Among the variations of the *MTHFR* gene, the polymorphisms of C677T and A1298C affect both nucleotide synthesis and DNA methylation. Compared with wild genotype (CC), the heterozygote (CT) and mutation homozygote (TT) lead to declines in enzyme activity of about 34% and 75%, respectively [4]. Homozygous carriers of the 1298C allele have a more moderate 30–40% reduction of the enzyme activity, but its function remains controversial.

Epidemiological research has reported that attention-deficit hyperactivity disorder (ADHD), bipolar disorder (BD) and schizophrenia (SCZ) are multimorbid conditions that are typically accompanied by cognitive advantages or deficits, suggesting that common biological mechanisms may underlie these phenotypes [5]. The complex neurodevelopment disorder ADHD affects around 5% of school-aged children [6], and 65% of them can be still affected when they are grown up, which has significant social, academic and occupational effects [7]. Its prevalence in adults is approximately 2.5% [8]. The etiology of ADHD is not fully understood and remains inconclusive. Family, twin and adoption studies have identified the impact of genetic variation on ADHD risk. Not only environment, such as maternal smoking, but genetic factors also play an important role. Molecular genetics research has gradually ascertained the inherited susceptible genes for ADHD. Recent investigations reported that the average heritability was estimated at 76% [9, 10] in childhood and 30–50% [11–13] or even higher in adulthood [14, 15].

Characterized by alternating episodes of depression and mania [16], BD is a serious common chronic mental illness with population prevalence of about 1–2% [17]. Although it is more common than previously thought, it has received less attention in terms of research than other major psychiatric disorders. Family, twin and adoption studies have identified the impact of genetic variation on the risk of BD [18]. Age at onset and polarity at onset are related to the indicators of BD severity. The patients at an earlier onset show an increased polygenic liability of psychiatric disorders [19]. Both ADHD and BD are neurodevelopmental disorders with onset in childhood and early adolescence, and common persistence in adulthood [20].

Affected by the mutual influence of multiple genetic and environmental factors, SCZ is a common mental disorder with heritability up to 80% [21]. Patients with SCZ experience higher mortality rates than the general population, especially due to suicide [22]. Large-scale epidemiological studies have consistently shown that infections, autoimmune diseases and atopic disorders are associated with increased risk of SCZ and that SCZ is associated with increased levels of immune markers at diagnosis [23].

Recent studies showed that *MTHFR* genetic polymorphism is related to neuropsychiatric diseases such as ADHD, BD and SCZ [24–27]. Polymorphisms of *MTHFR* C677T are likely to be associated with the risk of developing BD and SCZ and influence the age at onset of BD but not for SCZ [28]. A regression model found the TT genotype of the C677T locus was associated with the lowest global methylation. Moreover, the C677T allele might represent different liability according to gender [29]. However, some studies failed to find any association between *MTHFR* C667T polymorphism and risk of SCZ and BD [30, 31]. Due to the small number of studies and the limited sample size, conclusions are not clear.

Meta-analysis is a widely used statistical method in medical studies, particularly for topics that are being extensively studied with controversial results [32]. No meta-analysis has yet reported on association between *MTHFR* polymorphism and ADHD occurrence. One meta-analysis reported that the *MTHFR* C677T locus was significantly associated with BD in 2011 (sample size 29,502) [33]. There have been four meta-analyses concerning the association of SCZ [33–36]. The latest study found that *MTHFR* A1298C polymorphism was a risk factor for SCZ, which included 19 studies with 4049 cases and 5488 controls [36]. To better understand the role of *MTHFR* in the occurrence of psychiatric disorders, we conducted a meta-analysis of all published case-control studies exploring the associations between two common polymorphisms (677C>T and 1298A>C) of *MTHFR* and three psychiatric disorders: ADHD, BD and SCZ. This will provide a more comprehensive assessment of the association between this polymorphism and ADHD, BD and SCZ.

## Materials and methods

### Identification and eligibility of relevant studies

To identify eligible studies for inclusion in this meta-analysis, we searched the PubMed electronic database up to December 2021, without restriction on article type in English. The following keywords were used in the literature search: 5,10-methylenetetrahydrofolate reductase, MTHFR, and one of the following three words: ADHD, BD or SCZ. The selected studies met the following inclusion criteria: (1) case-control design, (2) including patients with one of the three diseases and (3) stating available allele or genotype frequencies. Of the studies with the same or overlapping data published by the same authors, the latest articles were selected. Major reasons for exclusion follow: (1) no control population, (2) duplicate of an earlier publication and (3) lack of usable genotype frequency data. If we needed to retrieve additional data that were not contained in the original report, we contacted the corresponding authors for additional details (e.g., allele or genotype frequencies or sample characteristics).

### Data extraction

Based on the inclusion criteria, two reviewers (Mao-ling Sun and Jun Yao) independently extracted information from all the included studies. Disagreements were resolved by discussion until the two reviewers reached a consensus. The following data were extracted from each study: first author’s family name, publication year, country and number of genotypes between cases and controls. To delineate potential moderating influences on the effects obtained from the case-control studies considered, we also included the following variables: (1) diagnostic criteria, (2) controls source, (3) mean age of cases and (4) proportion of males in the disease sample.

### Quality assessment

Two authors (Mao-ling Sun and Jun Yao) independently assessed the quality of the included studies according to the Newcastle Ottawa Scale (NOS) (www.ohri.ca/programs/clinical_epidemiology/oxfprd.asp). This scale consists of three components related to sample selection, comparability and ascertainment of exposure. A score of five or more was considered “high quality”; studies with scores from zero to four were assessed as “low quality”.

### Statistical analysis

Hardy–Weinberg equilibrium (HWE) in the genotype distribution of controls was tested using the chi-square goodness of fit. Odds ratios (ORs) with 95% confidence intervals (CIs) were calculated to measure the strength of the association between the target locus and the disease. Pooled effect sizes across studies were determined using five genetic models (allele contrast, homozygous codominant, heterozygous codominant, dominant and recessive) by a random effects model, which could reduce the bias due to the heterogeneity from multiple studies. The degree of heterogeneity between studies was determined by Q-statistic, with *p* > 0.05 indicating a lack of heterogeneity and *p* < 0.05 indicating heterogeneity. Moreover, I^2^ was calculated to quantify the apparent inconsistency; its conventional interpretation for existing heterogeneity is low (<25%), moderate (approximately 50%) and high (>75%). Additionally, Begg’s funnel plot and Egger’s test were used to evaluate publication bias.

Sensitivity analysis was performed to assess the potential influences of a single study on the pooled effect size. It was performed by omitting single studies one at a time for each meta-analysis to screen for significant alterations to pooled effect size.

All statistical tests were two-sided, with *p* < 0.05 considered significant. The meta-analysis was conducted using Stata version 16.0 software (Stata Corp., College Station, TX, USA).

## Results

**Fig 1:**
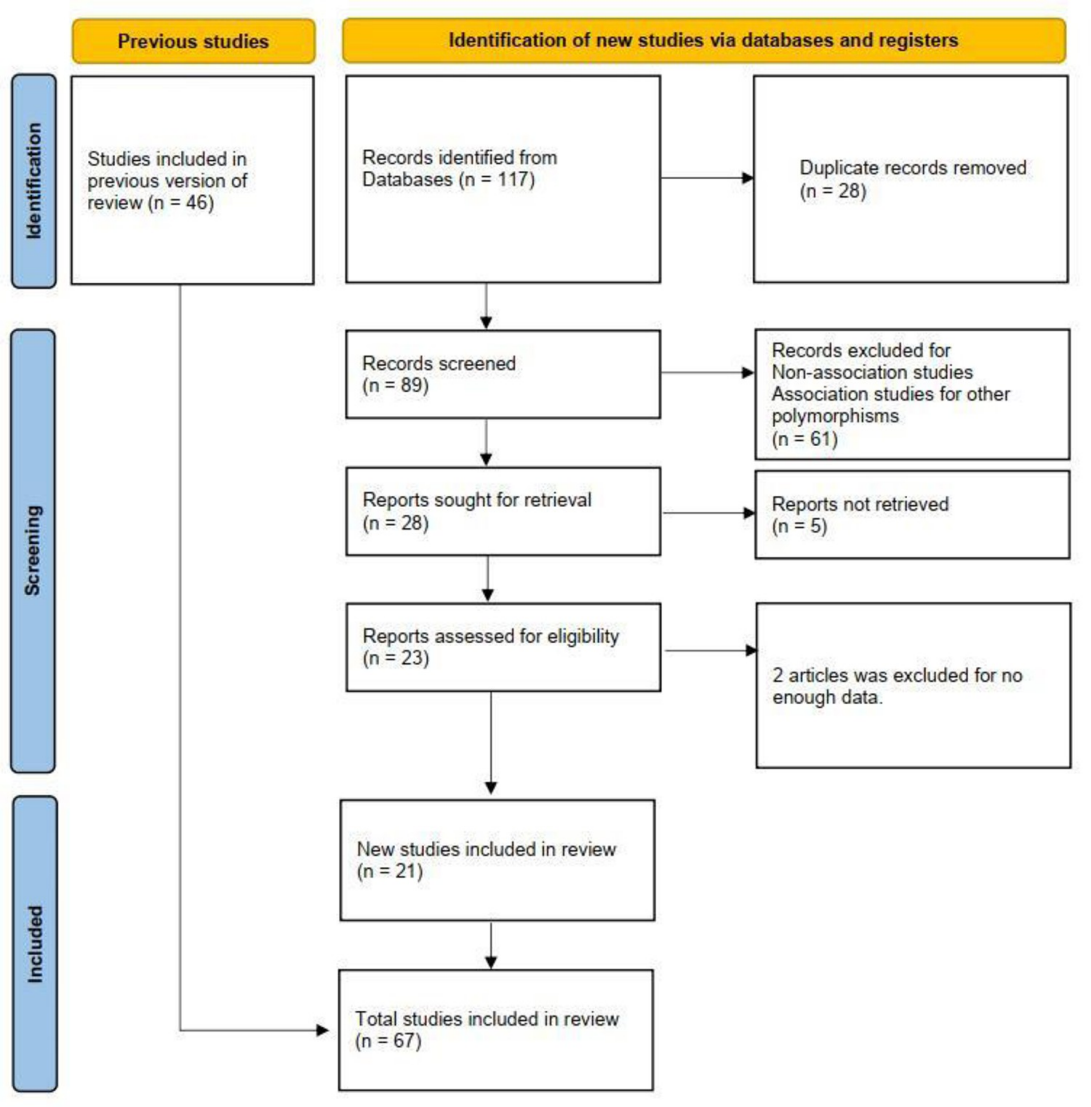
Flow of study identification, inclusion and exclusion.

After the removal of overlapping articles and those that did not meet the inclusion criteria (Fig 1), a total of five studies with ADHD including 434 cases and 670 controls [2, 37–40], 18 studies with BD including 4167 cases and 5901 controls [28–31, 41–53] and 44 studies with SCZ including 16,098 cases and 19913 controls were finally included in our meta-analysis [28–31, 42, 46, 47, 52, 54–76]. The key characteristics of the studies and NOS scale information are presented in Table 1. The NOS scale results showed that 66 studies were of high quality and one study was of low quality. Genotype and allele frequencies, HWE and sample size are given in Tables 2–4, respectively. Of the total of 67 studies, four showed significant deviations from HWE (*p* < 0.05).

**Table 1:** Baseline characteristics of qualified studies in this meta-analysis.

Author	Year	Country	Disease	Diagnostic criteria	Controls source	Mean age of cases (years)	Male (%)	NOS scores
Baykal	2019	Turkey	ADHD	DSM-V	hospital-based	8.70±2.77	78.1	5
Ergul	2012	Turkey	ADHD	DSM- IV	hospital-based	8.87±2.55	80	5
Gokcen	2011	Turkey	ADHD	DSM- IV	population-based	9.77±2.3	77.5	7
Krull	2008	U.S.	ADHD	-	-	8.70±2.77	78.1	4
Sadeghiyeh	2020	Aryan	ADHD	DSM- IV	population-based	8.13±1.34	82.2	7
Agnieszka	2014	Poland	BD	DSM-IV-TR	hospital-based	51±14	20	7
Arinami	1997	Japan	BD	DSM-IV	population-based	-	-	5
Arzaghi	2011	Iran	BD	DSM-IV	hospital-based	35±8	56.7	7
Chen	2009	China	BD	DSM-IV	population-based	36.6±7.2	-	7
El-Hadidy	2014	Egypt	BD	DSM-IV-TR	hospital-based	32.2±10.9	53.7	7
El-Hadidy	2013	Egypt	BD	DSM-IV	hospital-based	-	-	5
Ezzaher	2011	Tunisia	BD	DSM-IV	hospital-based	36±11.1	67.3	7
Jasson	2008	Norway	BD	DSM-IV	population-based	41±12.2	39.3	8
Kempisy	2006	Poland	BD	DSM-IV	population-based	44.5±13.5	52.5	8
Kempisy	2007	Poland	BD	DSM-IV	population-based	43.5±13.5	52.5	8
Kunugi	1998	Japan	BD	DSM-IV	population-based	47.9±13.6	37.1	8
Ozbek	2008	Turkey	BD	DSM-IV	population-based	40.55±12.33	37.6	8
Rahimi	2016	Iran	BD	DSM-IV-TR	hospital-based	35.4±12.3	50	7
Reif	2005	Germany	BD	DSM-IV	population-based	50	-	7
Sarah Woods	2010	UK, Canada	BD	DSM-IV	population-based	47.15±11.94	37.7	8
Tan	2004	Singapore	BD	DSM-IV	hospital-based	43.3±14	34.1	7
Wang	2015	China	BD	DSM-IV-TR	population-based	31.9±11.5	48	8
Zhao	2008	China	BD	DSM-IV	population-based	-	-	7
Gao	2020	China	SCZ	DSM-IV	population-based	47.8±10.2	81.8	8
Arinami	1997	Japan	SCZ	DSM-IV	-	-	-	5
Arzaghi	2011	Iranian	SCZ	DSM-IV	hospital-based	29±4	68.18	7
Betcheva	2009	Bulgaria	SCZ	DSM-IV	-	-	-	5
Bouaziz	2010	Tunisia	SCZ	DSM-IV-TR	population-based	36.0±9.0	100	8
El-Hadidy	2014	Egypt	SCZ	DSM-IV-TR	hospital-based	33.9±9.4	36.46	7
Feng	2009	China	SCZ	DSM-IV	hospital-based	31.7	40.65	7
Foroughmand.AM	2015	Iran	SCZ	DSM-IV-TR	population-based	43.3±11.3	58.5	7
Garcia-Miss Mdel	2010	Mexico	SCZ	DSM-IV-TR	population-based	38±9	70.48	8
Hei	2014	China	SCZ	DSM-IV	hospital-based	27±12	54.6	7
Jonsson	2008	Norway	SCZ	DSM-IV	population-based	36.6±9.8	53.99	8
Jonsson	2008	Denmark	SCZ	ICD-10	population-based	44.4±11.2	57.52	8
Jonsson	2008	Sweden	SCZ	DSM-III-R	population-based	55.7±15.6	62.02	8
Joober	2000	Canada	SCZ	DSM-IV	population-based	-	-	7
Kang.HJ	2010	Korean	SCZ	DSM-IV	population-based	38.32±3.93	54	8
Kempisty	2007	Poland	SCZ	DSM-IV	population-based	43.5±13.5	52.5	8
Kempisty	2006	Poland	SCZ	DSM-IV	population-based	10.39	50.5	8
Kim	2011	Korean	SCZ	DSM-IV	hospital-based	32.89±7.76	66.17	7
Kontis.D	2013	Greece	SCZ	DSM-IV	population-based	42.91±10	64.44	8
Kunugi	1998	Japan	SCZ	DSM-IV	population-based	42.2±12.8	48.69	8
Lajin.B	2012	Syrian	SCZ	DSM-IV	hospital-based	37±10	70.59	7
Lee	2006	Korea	SCZ	DSM-IV	population-based	-	42.55	7
Misiak.B	2016	Poland	SCZ	DSM-IV,ICD-10	hospital-based	27.2±6.5	55.6	7
Muntjewerff	2008	Netherlands	SCZ	DSM-IV	population-based	41±14	73	8
Muntjewerff.JW	2011	Netherlands	SCZ	DSM-IV	hospital-based	39±14	75	7
Nishi	2014	Japan	SCZ	DSM-IV	population-based	54.6±14.9	48.6	8
Nishi	2014	Japan	SCZ	DSM-IV	population-based	46.5±15.8	58.8	8
Philibert	2006	USA	SCZ	DSM-IV	population-based	-	63.59	7
Roffman	2008	USA	SCZ	DSM-IV-TR	population-based	-	-	7
Sazci	2005	Turkey	SCZ	DSM-IV	population-based	41.22±9.43	56.57	8
Sazci	2003	Turkey	SCZ	DSM-IV	hospital-based	42.22±13.17	90	8
Tan	2004	Singapore	SCZ	DSM-IV	hospital-based	55.2±10.3	75.85	7
Tsutsumi	2011	Japan	SCZ	DSM-IV	population-based	47.2	53.51	8
Vilella	2005	Spain	SCZ	ICD-9	population-based	55.4±12.1	59.49	8
Virgos	1999	Spain	SCZ	ICD-9	-	-	-	5
Wan	2019	China	SCZ	DSM-IV	hospital-based	29.2±11.6	49.2	7
Wan	2019	China	SCZ	DSM-IV	hospital-based	14.07	52.6	7
Ye	2010	China	SCZ	DSM-IV	hospital-based	32.4±6.3	42.31	7
Yu	2004	China	SCZ	DSM-III-R	population-based	-	-	7
Yu	2004	Scotland	SCZ	DSM-III-R	population-based	-	-	7
Zhang	2013	China	SCZ	DSM-IV	population-based	31.2±9.9	54	8
Zhang	2012	China	SCZ	DSM-IV	hospital-based	31±9	55.74	7
Zhang	2010	China	SCZ	DSM-IV	hospital-based	32.1±9.7	58.05	7
Zhilyaeva.TV	2018	Russia	SCZ	ICD-10	population-based	42.22±12.7	55.4	8

Note: Male (%) = the proportion of males in the case samples.

**Table 2:** Distribution of genotype and allele frequencies of the *MTHFR* 677C>T and 1298A>C polymorphisms in ADHD patients.

	677C>T genotype							1298A>C genotype								
	Cases, n			Controls, n				Cases, n			Controls, n				Sample size	
Author	CC	CT	TT	CC	CT	TT	*P*_HWE_	AA	AC	CC	AA	AC	CC	*P*_HWE_	case	control
Bayhal	24	34	6	14	20	6	0.7921	26	32	6	16	22	2	0.1088	64	40
Ergul	44	47	9	154	125	21	0.5195	37	53	10	121	133	46	0.3477	100	300
Gokcen	22	18	0	15	15	0	0.0679	9	31	0	14	16	0	**0.0464**	40	30
Krull	6	10	0	40	40	0	**0.0029**	5	11	0	37	43	0	**0.0010**	16	80
Sadeghiyeh	61	94	59	72	99	49	0.1816	45	98	71	59	107	54	0.6913	214	220

Note: *P*_HWE_ represents the *P* value of Hardy-Weinberg equilibrium test in the genotype distribution of controls.

**Table 3:** Distribution of genotype and allele frequencies of the *MTHFR* 677C>T and 1298A>C polymorphisms in bipolar disorder patients.

	677C > T Genotype							1298A > C Genotype								
	Cases, n			Controls, n				Cases, n			Controls, n				Sample size	
Author	CC	CT	TT	CC	CT	TT	*P*_HWE_	AA	AC	CC	AA	AC	CC	*P*_HWE_	case	control
Agnieszka	51	50	11	66	85	16	0.1266								112	167
Arinami	15	20	5	154	214	51	0.0743								40	419
Arzaghi	52	34	4	54	38	2	0.1096								90	94
Chen	178	231	92	153	235	73	0.2718								501	461
El-Hadidy	46	70	18	114	30	5	0.1026								134	149
El-Hadidy	42	40	8	72	30	6	0.2390								90	108
Ezzaher	41	40	11	94	62	14	0.4106								92	170
Jasson	58	49	10	80	75	22	0.5008	47	56	12	82	79	16	0.6243	232	354
Kempisy	108	73	19	210	79	11	0.3027								200	300
Kempisy								99	78	23	185	105	10	0.2903	200	300
Kunugi	41	74	28	95	129	34	0.3416								143	258
Ozbek	104	76	17	116	97	25	0.4846	91	84	22	113	101	24	0.8377	394	476
Rahimi	69	67	14	81	62	5	0.0934								150	148
Reif	48	34	10	75	80	21	0.9623	30	47	15	75	96	13	**0.0163**	184	360
Sarah Woods	362	386	98	642	719	216	0.5158								846	1577
Tan	99	60	8	80	33	7	0.1645								167	120
Wang	287	206	38	215	119	33	**0.0073**								531	367
Zhao	12	28	21	18	40	15	0.4036								61	73

Note: *P*_HWE_ represents the *P* value of Hardy-Weinberg equilibrium test in the genotype distribution of controls.

**Table 4:** Distribution of genotype and allele frequencies of the *MTHFR* 677C>T and 1298A>C polymorphisms in schizophrenia patients.

	677C > T Genotype							1298A > C Genotype								
	Cases, n			Controls, n				Cases, n			Controls, n				Sample size	
Author	CC	CT	TT	CC	CT	TT	*P*_HWE_	AA	AC	CC	AA	AC	CC	*P*_HWE_	case	control
Arinami	96	138	63	154	214	51	0.0743								297	419
Arzaghi	35	27	4	54	38	2	0.1096								66	94
Betcheva	76	85	24	84	76	22	0.4565	91	72	18	80	79	24	0.5213	366	365
Bouaziz	18	4	3	19	5	1	0.3969								25	25
El-Hadidy	48	28	20	72	30	6	0.2390								96	108
Feng	17	67	39	40	65	18	0.3084								123	123
Foroughmand.AM	104	76	20	123	64	13	0.2437	60	89	51	65	108	27	0.0885	400	400
Gao	298	344	123	145	202	70	0.9802								765	417
Garcia-Miss Mdel	29	45	31	22	54	31	0.8642								105	107
Hei	17	65	48	24	38	18	0.6898								130	80
Jonsson	75	70	18	80	75	22	0.5008	89	60	14	82	79	16	0.6243	326	354
Jonsson	200	177	42	490	413	103	0.2494	184	186	48	462	419	123	0.0664	837	2010
Jonsson	137	104	17	156	113	24	0.5809	110	113	35	122	129	42	0.4062	516	586
Joober	30	52	23	41	36	13	0.2783								105	90
Kang.HJ	125	176	59	130	158	60	0.3168	248	105	7	239	100	9	0.7026	720	696
Kempisty	109	74	17	185	105	10	0.2903								200	300
Kempisty	113	68	19	210	79	11	0.3027								200	300
Kim	62	101	38	112	167	71	0.5440								201	350
Kontis.D	40	37	13	21	22	12	0.1868								90	55
Kunugi	121	168	54	95	129	34	0.3416								343	258
Lajin.B	47	26	12	58	58	10	0.3879	32	38	15	65	48	13	0.3592	170	252
Lee	74	128	33	99	115	21	0.1257	157	71	7	145	77	14	0.3824	470	471
Misiak.B	64	52	16	71	53	22	**0.0280**	55	64	13	55	72	19	0.5445	264	292
Muntjewerff	110	111	31	205	165	35	0.8261								252	405
Muntjewerff.JW	334	319	86	405	389	92	0.9213								739	886
Nishi	220	309	92	174	239	73	0.5380								621	486
Nishi	417	530	202	1,072	1,260	410	0.2074								1149	2742
Philibert	107	83	16	176	137	46	**0.0212**								206	359
Roffman	41	27	11	35	32	8	0.8652								79	75
Sazci	144	115	38	161	156	24	0.0926	130	129	38	159	155	27	0.2005	594	682
Sazci	59	49	22	106	103	17	0.2361	57	59	14	114	93	19	0.9957	260	452
Tan	136	84	16	80	33	7	0.1645								236	120
Tsutsumi	160	184	69	138	183	64	0.8004								413	385
Vilella	58	75	25	85	110	39	0.7360	76	68	14	124	97	13	0.2858	316	468
Virgos	81	98	31	79	106	33	0.7928								210	218
Wan	45	122	75	71	113	50	0.6869	174	63	5	171	58	5	0.9749	484	468
Wan	24	47	26	24	43	25	0.5323	66	29	2	69	22	1	0.6034	194	184
Ye	12	58	34	14	32	10	0.2658								104	56
Yu	91	96	43	85	126	40	0.5543	177	209	40	292	272	64	0.9552	656	879
Yu	199	186	41	306	260	62	0.5351	130	78	22	154	81	16	0.2350	656	879
Zhang	166	450	384	213	505	318	0.6297								1000	1036
Zhang	96	113	26	52	45	5	0.2248								235	102
Zhang								230	127	22	260	108	12	0.8478	379	380
Zhilyaeva.TV	245	212	43	280	188	31	0.9406								500	499

Note: *P*_HWE_ represents the *P* value of Hardy-Weinberg equilibrium test in the genotype distribution of controls.

### Association between MTHFR 667C>T and ADHD

**Fig 2:**
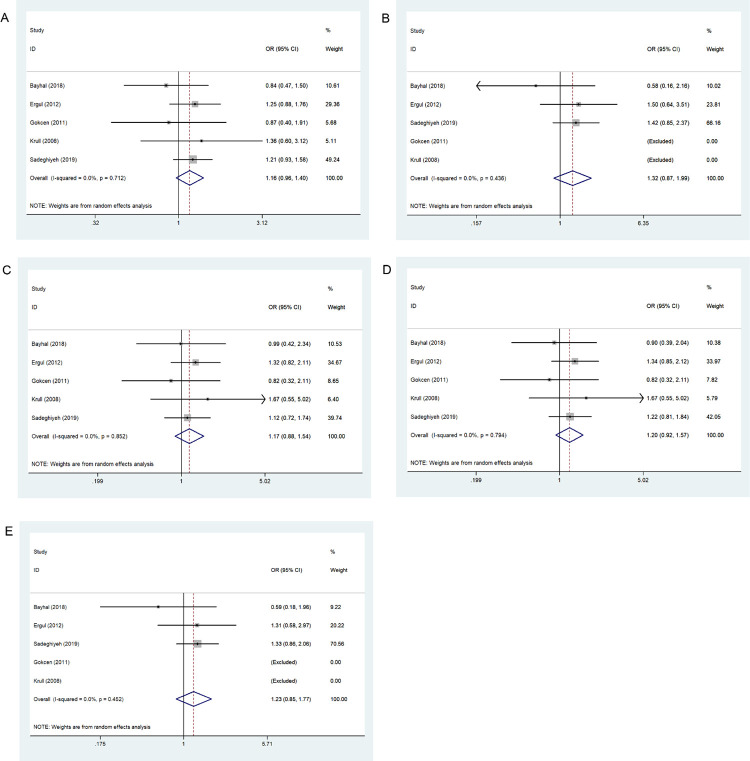
Forest plot of the association between 667C>T variation and risk of ADHD in the five genetic models: A, allele contrast; B, homozygous codominant; C, heterozygous codominant; D, dominant; and E, recessive.

Table 5 and Fig 2 show results generated for five genetic models evaluating the association between 667C>T variation and ADHD risk under a random effects model. Results indicated no association between 677C>T locus and ADHD occurrence.

**Table 5:** Summarized ORs with 95% CIs for the association between *MTHFR* polymorphisms and ADHD.

Polymorphism	Genetic model	n	Statistical model	OR	95% CI	*p*_z_	I^2^(%)	*p*_h_	*p*_e_
677C>T									
	Allele contrast	5	Random	1.161	0.962–1.400	0.119	0.0	0.712	0.367
	Homozygous codominant	3	Random	1.317	0.870–1.993	0.193	0.0	0.436	0.427
	Heterozygous codominant	5	Random	1.168	0.883–1.543	0.277	0.0	0.852	0.779
	Dominant	5	Random	1.205	0.924–1.571	0.169	0.0	0.794	0.544
	Recessive	3	Random	1.229	0.852–1.774	0.270	0.0	0.452	0.401
1298A>C									
	Allele contrast	5	Random	1.206	1.003–1.450	0.047	0.0	0.453	0.681
	Homozygous codominant	3	Random	1.255	0.650–2.420	0.497	44.1	0.167	0.873
	Heterozygous codominant	5	Random	1.321	0.987–1.767	0.061	0.0	0.428	0.352
	Dominant	5	Random	1.337	1.012–1.766	0.041	0.0	0.442	0.337
	Recessive	3	Random	1.132	0.558–2.297	0.731	59.0	0.087	0.850

Note: n, the number of studies; *p*_z_, *P* value for association test; *p*_h_, *p* value for heterogeneity test; *p*_e_, *p* value for publication bias test.

### Association between MTHFR 1298A>C and ADHD

**Fig 3:**
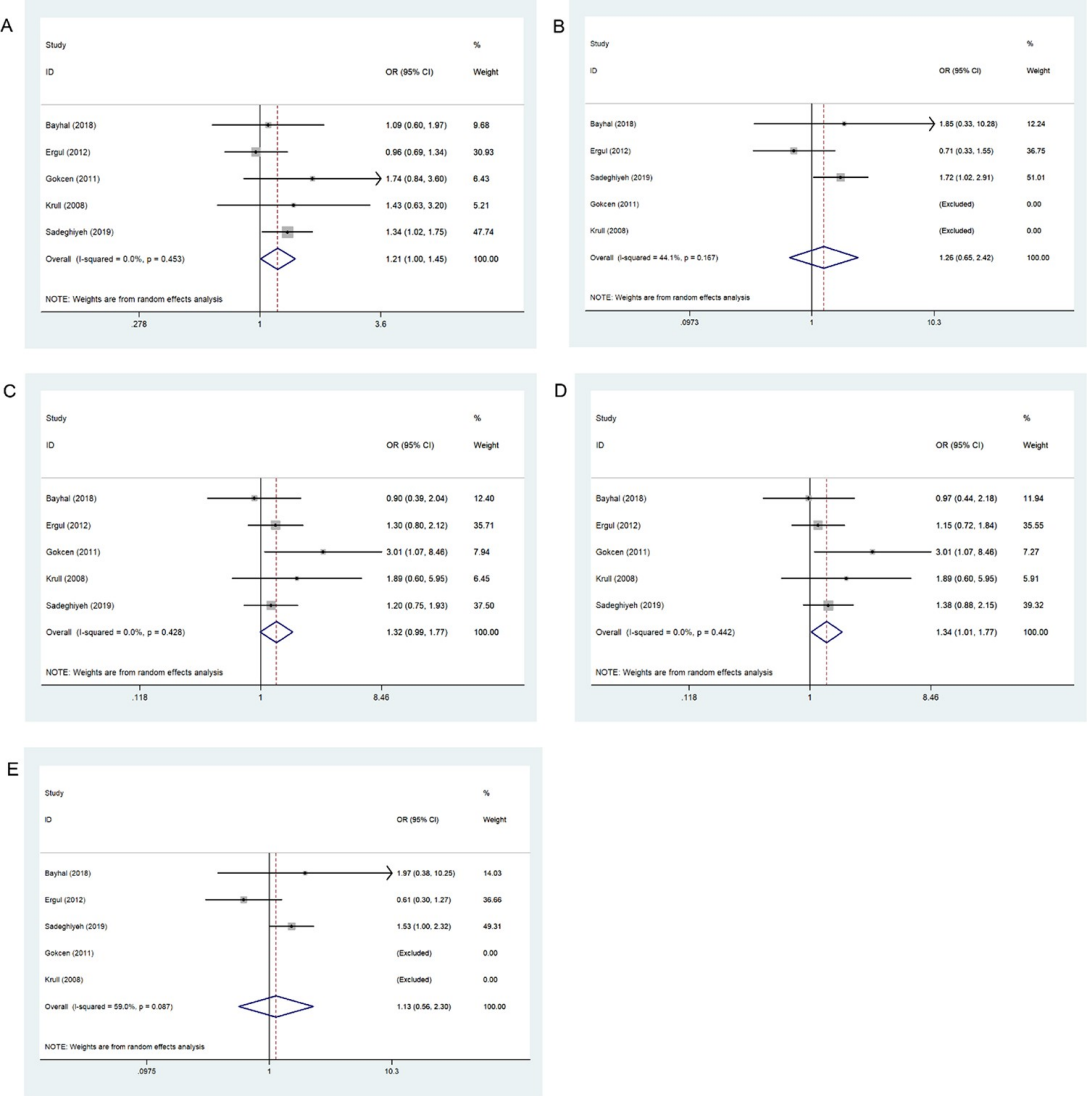
Forest plot of the associations between 1298A>C variation and risk of ADHD in the five genetic models: A, allele contrast; B, homozygous codominant; C, heterozygous codominant; D, dominant; and E, recessive.

Table 5 and Fig 3 show results for five genetic models evaluating associations between 1298A>C variation and ADHD risk under a random effects model. Results showed an association between 1298A>C and ADHD occurrence as a risk factor in the allele contrast (*p* = 0.047, OR = 1.206, 95% CI = 1.003–1.450) and the dominant models (*p* = 0.041, OR = 1.337, 95% CI = 1.012–1.766).

### Association between MTHFR 667C>T and BD

**Fig 4:**
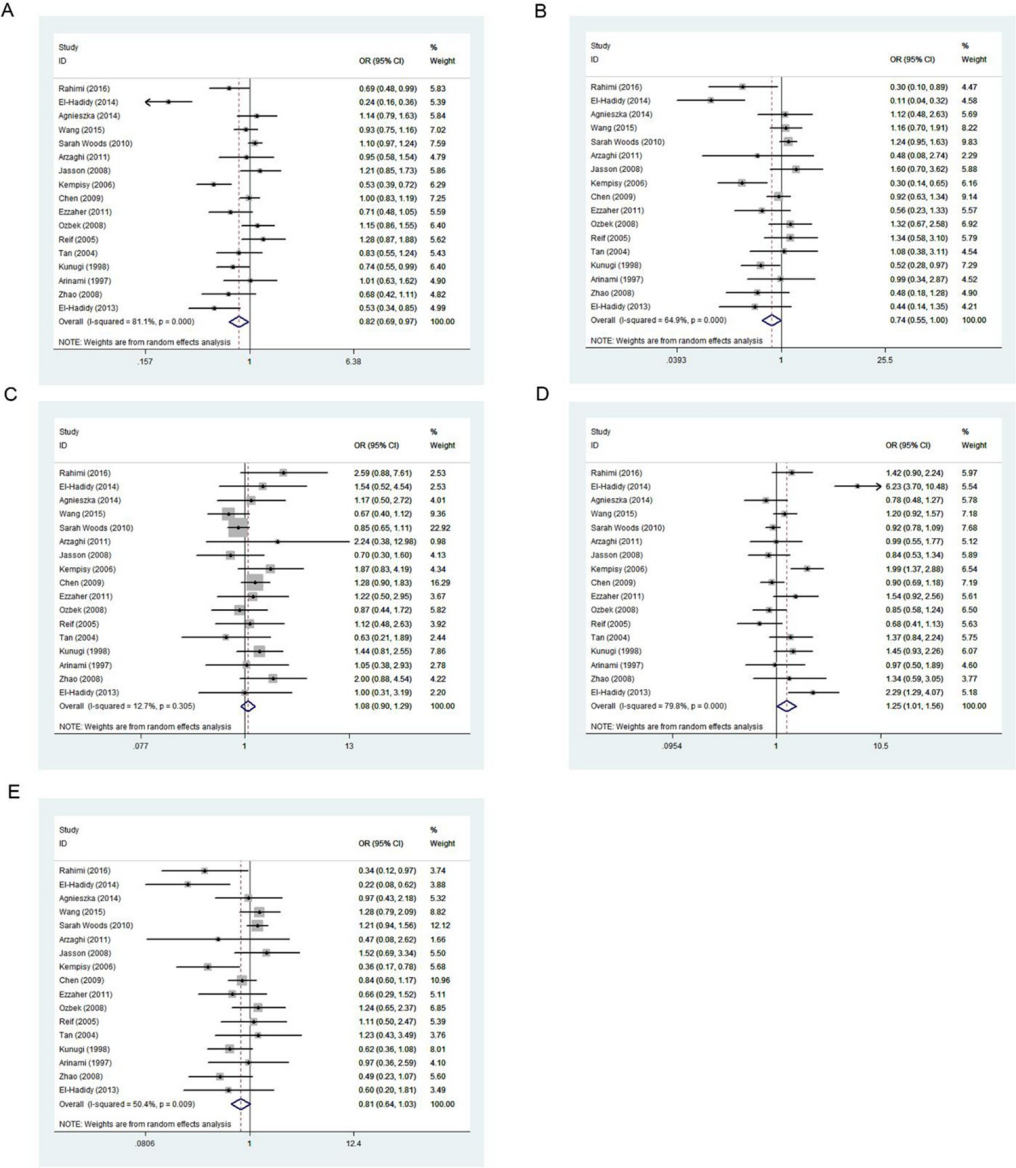
Forest plot of the association between 667C>T variation and risk of bipolar disorder in the five genetic models: A, allele contrast; B, homozygous codominant; C, heterozygous codominant; D, dominant; and E, recessive.

Table 6 and Fig 4 show the results for five genetic models evaluating the association between 667C>T variation and BD risk under a random effects model. The results indicated an association between 677C>T locus and BD occurrence as a protective factor in the allele contrast model (*p* = 0.024, OR = 0.822, 95% CI = 0.693–0.974) and as a risk factor in the dominant model (*p* = 0.044, OR = 1.254, 95% CI = 1.006–1.562).

**Table 6:** Summarized ORs with 95% CIs for the association between *MTHFR* polymorphisms and bipolar disorder.

Polymorphism	Genetic model	n	Statistical model	OR	95% CI	*p*_z_	I^2^(%)	*p*_h_	*p*_e_
677C>T									
	Allele contrast	17	Random	0.822	0.693–0.974	0.024	81.1	0.000	0.058
	Homozygous codominant	17	Random	0.744	0.553–1.001	0.050	64.9	0.000	0.025
	Heterozygous codominant	17	Random	1.079	0.905–1.287	0.396	12.7	0.305	0.128
	Dominant	17	Random	1.254	1.006–1.562	0.044	79.8	0.000	0.138
	Recessive	17	Random	0.810	0.640–1.026	0.080	50.4	0.009	0.036
1298A>C									
	Allele contrast	4	Random	0.756	0.602–0.950	0.017	50.8	0.107	0.991
	Homozygous codominant	4	Random	0.493	0.259–0.937	0.031	64.0	0.040	0.436
	Heterozygous codominant	4	Random	2.030	1.068–3.862	0.031	64.0	0.040	0.436
	Dominant	4	Random	1.326	1.075–1.636	0.008	0.0	0.409	0.941
	Recessive	4	Random	0.541	0.300–0.977	0.042	61.4	0.051	0.413

Note: n, the number of studies; *p*_z_, *P* value for association test; *p*_h_, *p* value for heterogeneity test; *p*_e_, *p* value for publication bias test.

### Association between MTHFR 1298A>C and BD

**Fig 5:**
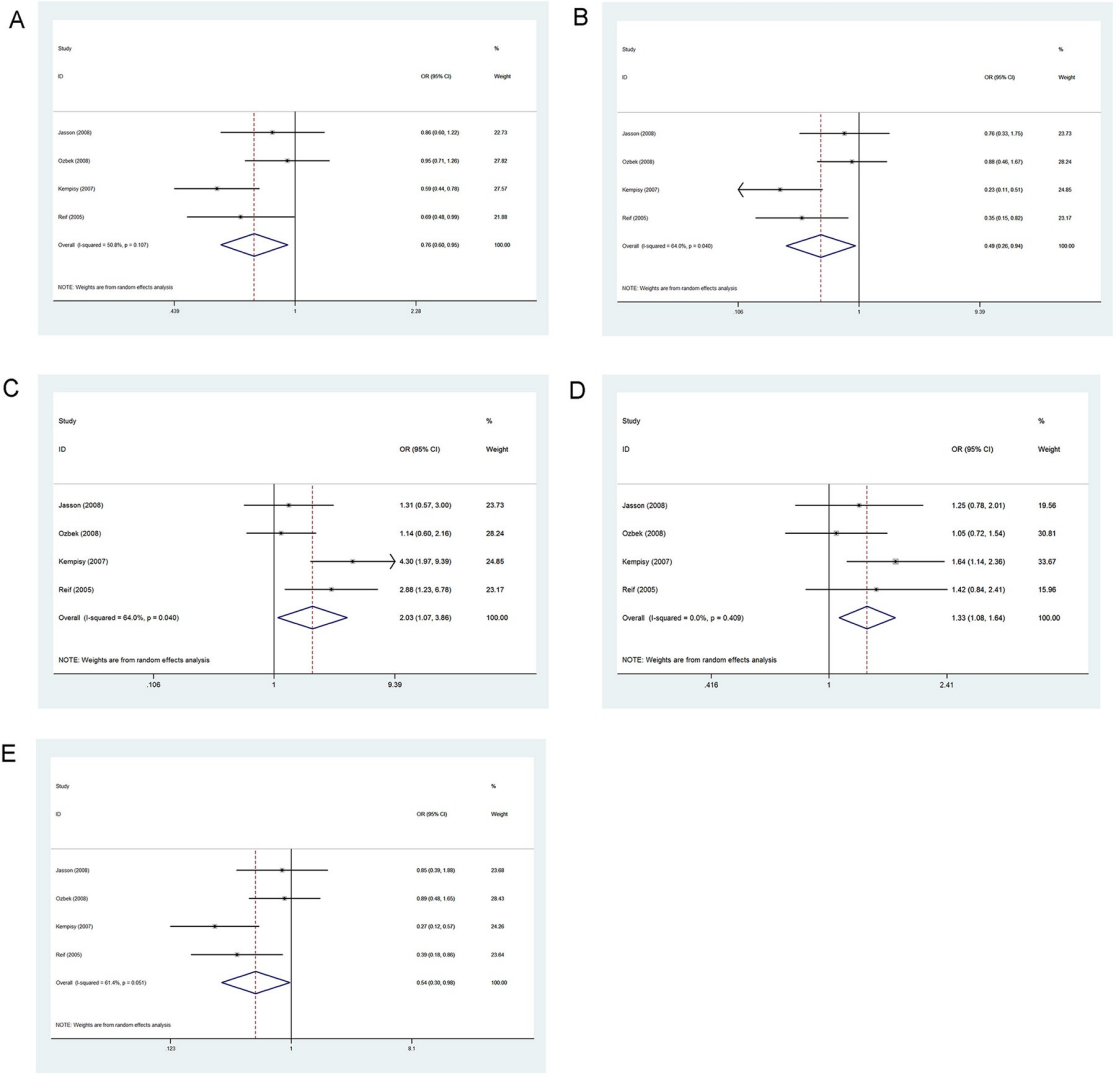
Forest plot of the associations between 1298A>C variation and risk of bipolar disorder in the five genetic models: A, allele contrast; B, homozygous codominant; C, heterozygous codominant; D, dominant; and E, recessive.

Table 6 and Fig 5 show the results for five genetic models evaluating associations between 1298A>C variation and BD risk under a random effects model. Our results showed an association between 1298A>C and BD occurrence as a protective factor in the allele contrast (*p* = 0.017, OR = 0.756, 95% CI = 0.602–0.950), homozygous codominant (*p* = 0.031, OR = 0.493, 95% CI = 0.259–0.937) and recessive models (*p* = 0.042, OR = 0.541, 95% CI = 0.300–0.977). However, the *MTHFR* 1298A>C increased the BD occurrence in the heterozygous codominant (*p* = 0.031, OR = 2.030, 95% CI = 1.068–3.862) and dominant models (*p* = 0.008, OR = 1.326, 95% CI = 1.075–1.636).

### Association between MTHFR 667C>T and SCZ

**Fig 6:**
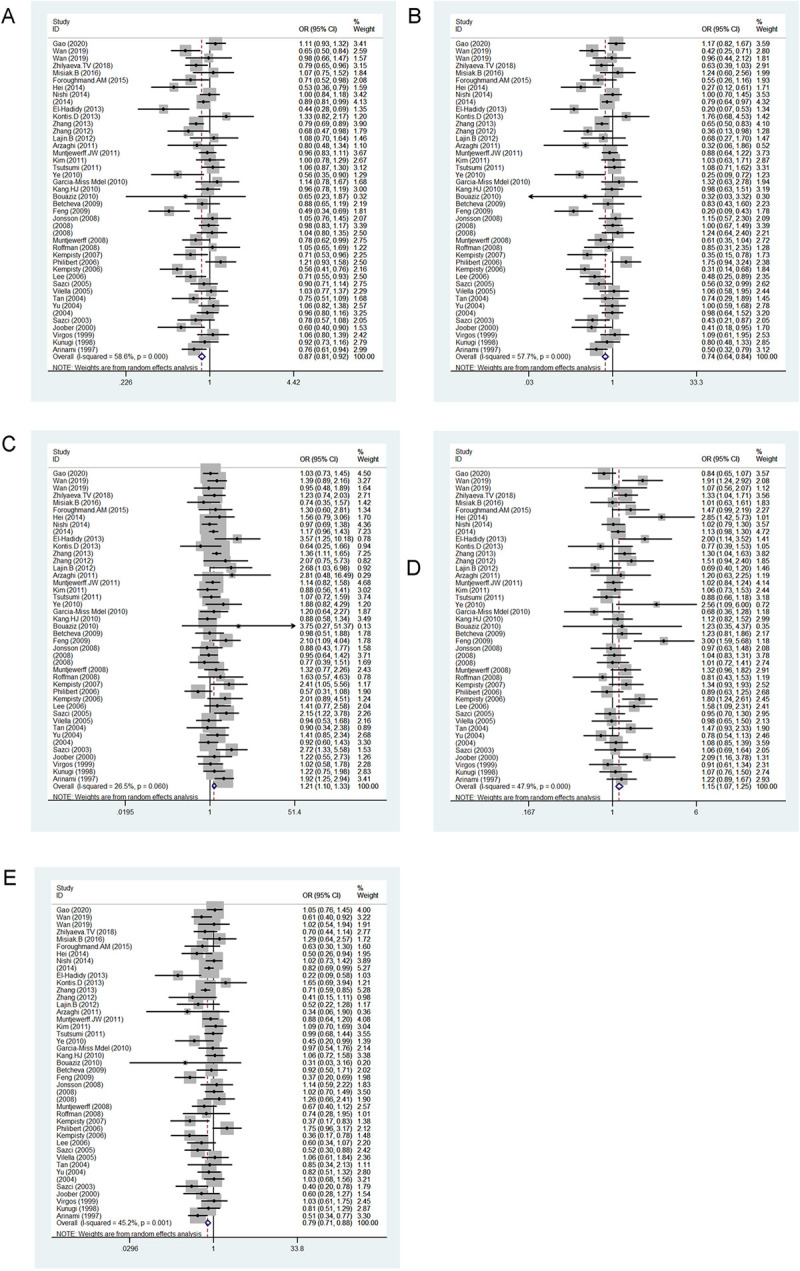
Forest plot of the association between 667C>T variation and risk of schizophrenia in the five genetic models: A, allele contrast; B, homozygous codominant; C, heterozygous codominant; D, dominant; and E, recessive.

Table 7 and Fig 6 show the results for five genetic models evaluating the association between 667C>T variation and SCZ risk under a random effects model. The results indicated an association between 677C>T locus and SCZ occurrence as a protective factor in the allele contrast (*p* < 0.001, OR = 0.867, 95% CI = 0.815–0.923), homozygous codominant (*p* < 0.001, OR = 0.735, 95% CI = 0.643–0.841) and recessive models (*p* < 0.001, OR = 0.787, 95% CI = 0.707–0.876) and as a risk factor in the heterozygous codominant (*p* < 0.001, OR = 1.211, 95% CI = 1.100–1.333) and dominant models (*p* < 0.001, OR = 1.153, 95% CI = 1.066–1.246).

**Table 7:** Summarized ORs with 95% CIs for the association between *MTHFR* polymorphisms and schizophrenia.

Polymorphism	Genetic model	n	Statistical model	OR	95% CI	*p*_z_	I^2^(%)	*p*_h_	*p*_e_
677C>T									
	Allele contrast	43	Random	0.867	0.815–0.923	0.000	58.6	0.000	0.135
	Homozygous codominant	43	Random	0.735	0.643–0.841	0.000	57.7	0.000	0.055
	Heterozygous codominant	43	Random	1.211	1.100–1.333	0.000	26.5	0.060	0.196
	Dominant	43	Random	1.153	1.066–1.246	0.000	47.9	0.000	0.104
	Recessive	43	Random	0.787	0.707–0.876	0.000	45.2	0.001	0.136
1298A>C									
	Allele contrast	17	Random	0.925	0.845–1.013	0.094	41.4	0.038	0.851
	Homozygous codominant	17	Random	0.852	0.691–1.052	0.136	35.4	0.074	0.833
	Heterozygous codominant	17	Random	1.113	0.926–1.338	0.254	18.4	0.239	0.756
	Dominant	17	Random	1.085	0.984–1.196	0.103	18.2	0.240	0.788
	Recessive	17	Random	0.867	0.711–1.057	0.158	34.0	0.084	0.800

Note: n, the number of studies; *p*_z_, *P* value for association test; *p*_h_, *p* value for heterogeneity test; *p*_e_, *p* value for publication bias test.

### Association between MTHFR 1298A>C and SCZ

**Fig 7:**
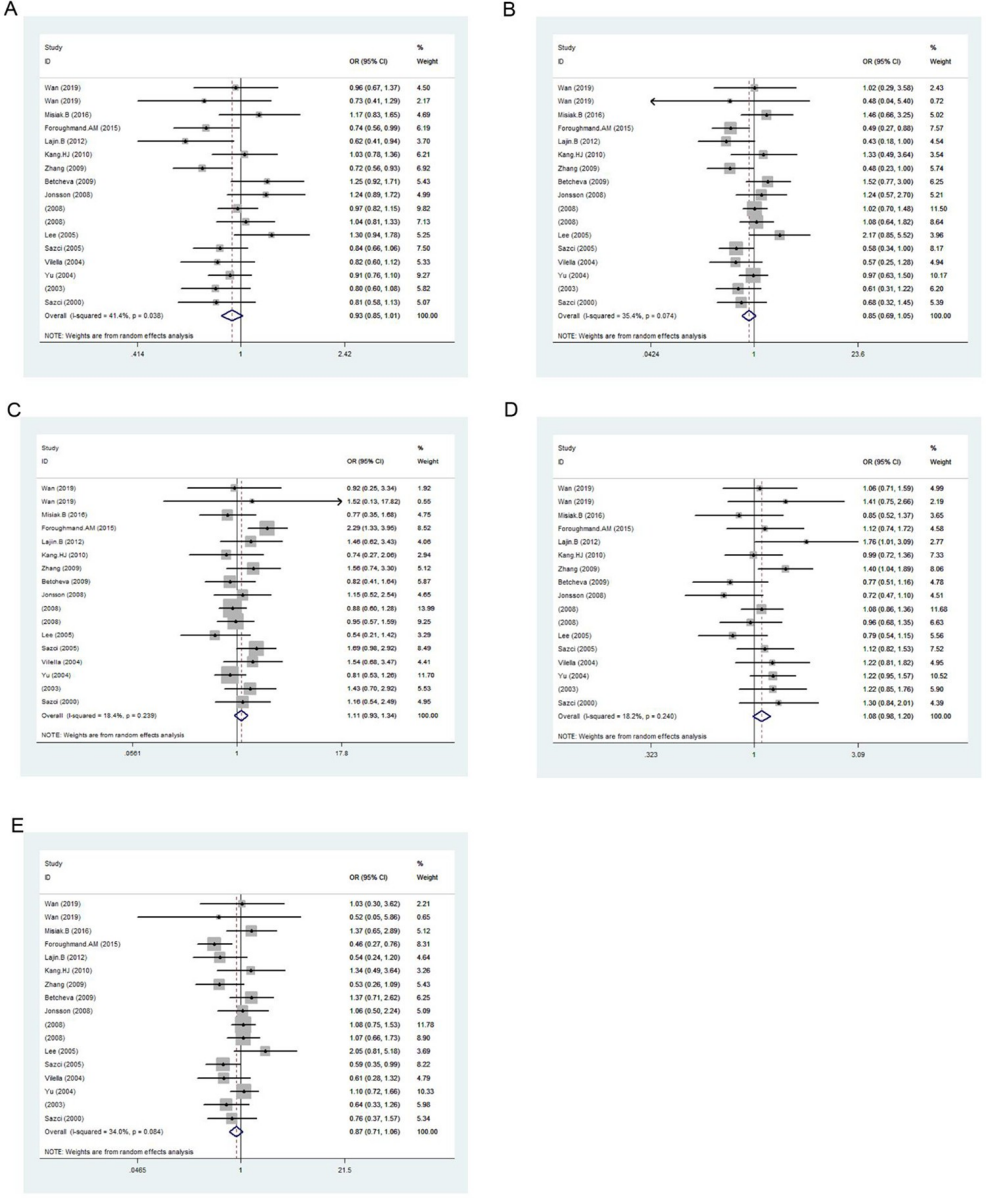
Forest plot of the associations between 1298A>C variation and risk of schizophrenia in the five genetic models: A, allele contrast; B, homozygous codominant; C, heterozygous codominant; D, dominant; and E, recessive.

Table 7 and Fig 7 show the results for five genetic models evaluating associations between 1298A>C variation and SCZ risk under a random effects model. The results showed no association between 1298A>C and SCZ occurrence in the five genetic models.

### Sensitivity analysis

We examined the influence of individual studies in the pooled ORs for 667C>T and 1298A>C loci via sensitivity analysis involving omitting each study in each genetic model; the results did not change. This indicates that our results were statistically robust for all five genetic models examining associations between *MTHFR* polymorphisms and susceptibility to ADHD, BD and SCZ.

### Publication bias

**Fig 8:**
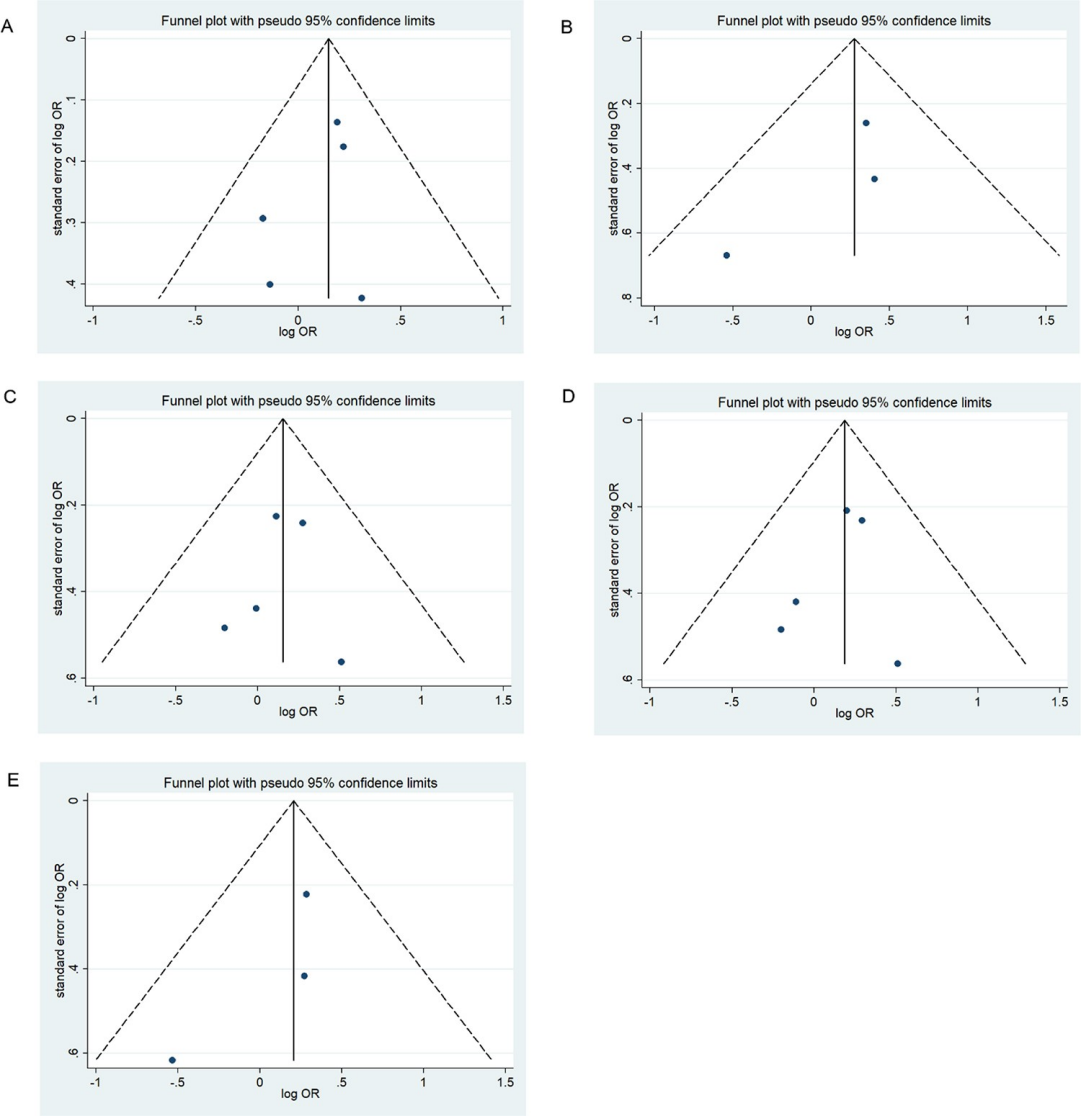
Funnel plot analysis depicting publication bias in the association between *MTHFR* 677C>T polymorphism and ADHD in the five genetic models: A, allele contrast; B, homozygous codominant; C, heterozygous codominant; D, dominant; and E, recessive.

**Fig 9:**
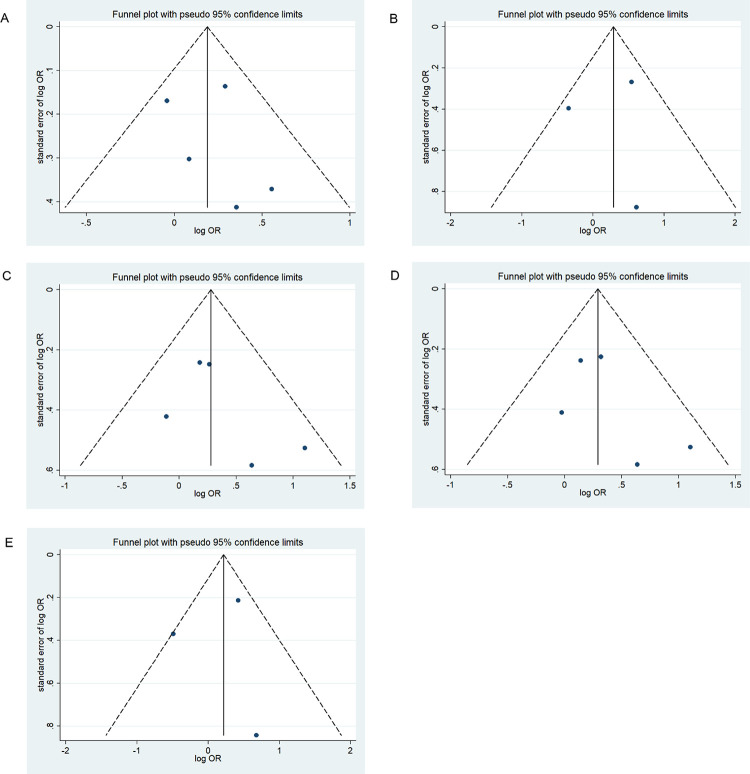
Funnel plot analysis depicting publication bias in the association between *MTHFR 1298A>C* polymorphism and ADHD in the five genetic models (A, allele contrast; B, homozygous codominant; C, heterozygous codominant; D, dominant; and E, recessive).

**Fig 10:**
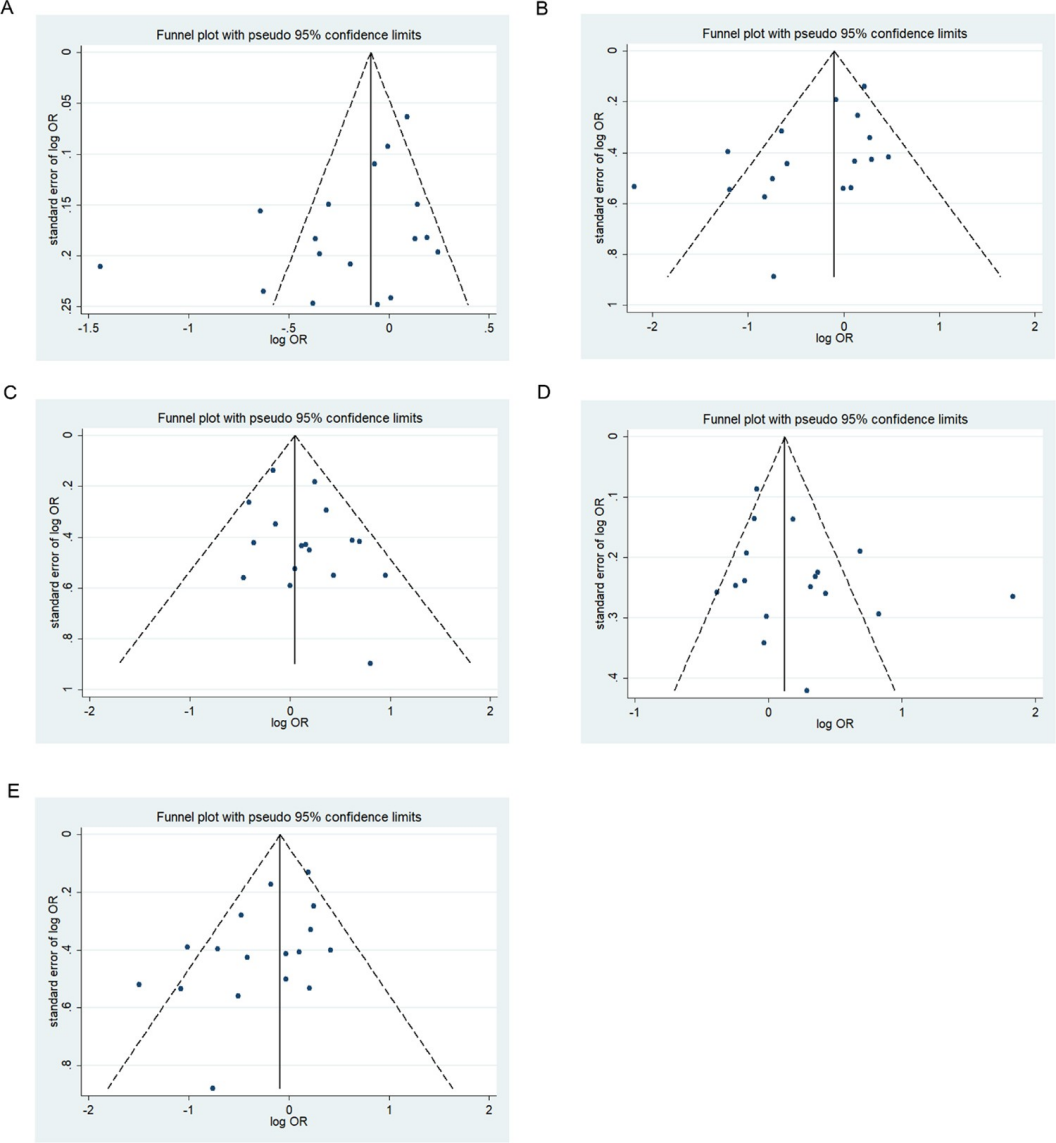
Funnel plot analysis depicting publication bias in the association between *MTHFR 677C>T* polymorphism and bipolar disorder in the five genetic models (A, allele contrast; B, homozygous codominant; C, heterozygous codominant; D, dominant; and E, recessive).

**Fig 11:**
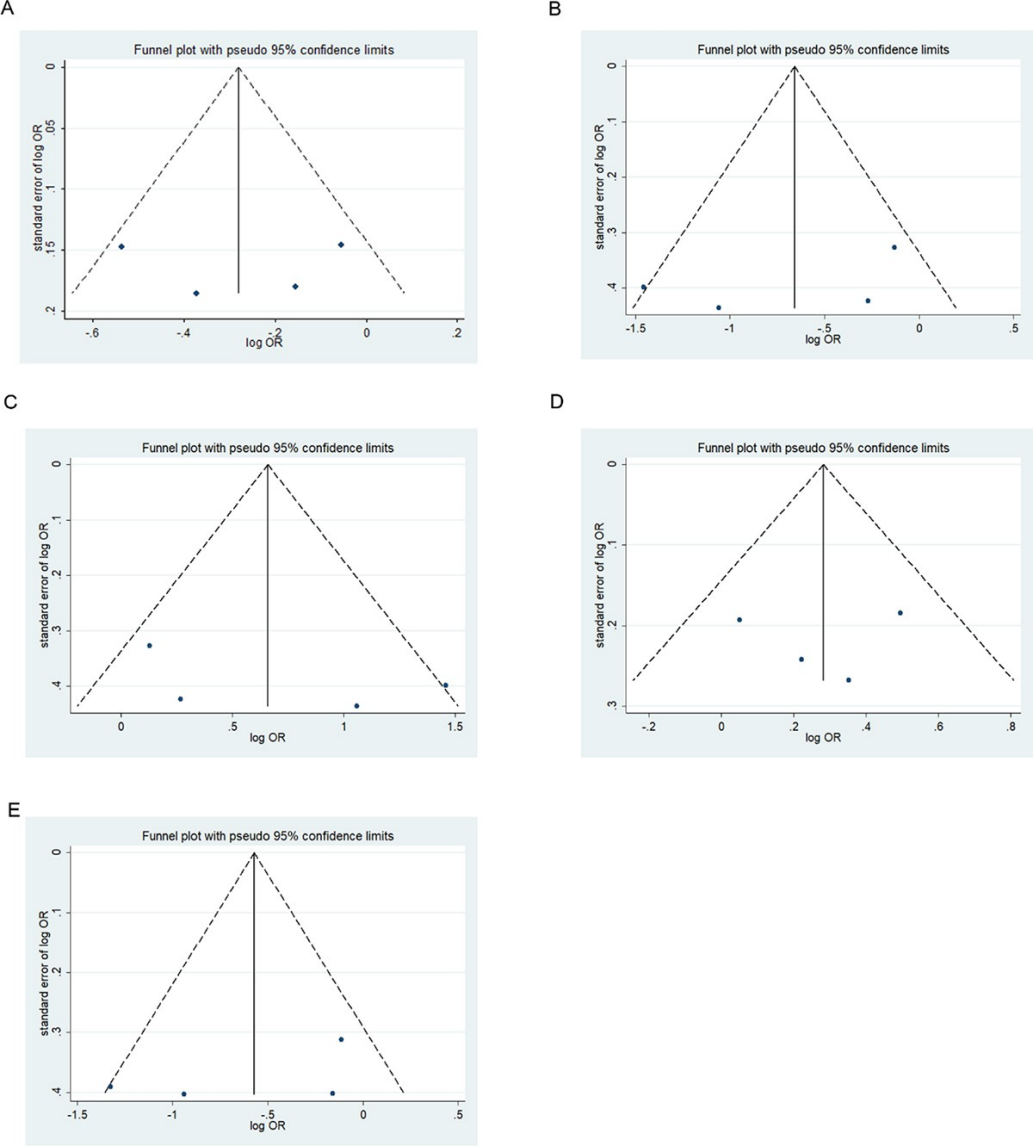
Funnel plot analysis depicting publication bias in the association between *MTHFR 1298A>C* polymorphism and bipolar disorder in the five genetic models (A, allele contrast; B, homozygous codominant; C, heterozygous codominant; D, dominant; and E, recessive).

**Fig 12:**
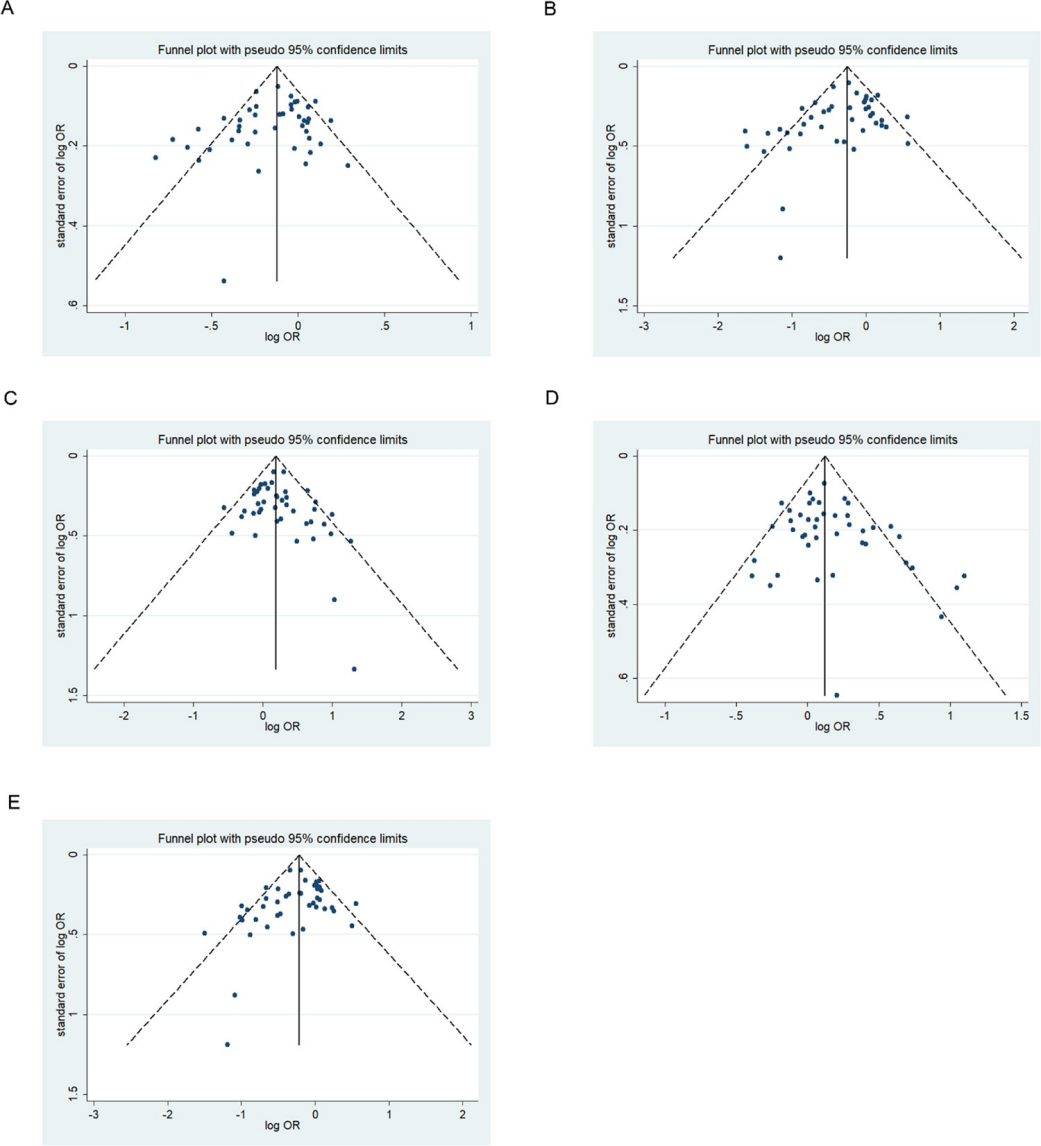
Funnel plot analysis depicting publication bias in the association between *MTHFR 677C>T* polymorphism and schizophrenia in the five genetic models (A, allele contrast; B, homozygous codominant; C, heterozygous codominant; D, dominant; and E, recessive).

**Fig 13:**
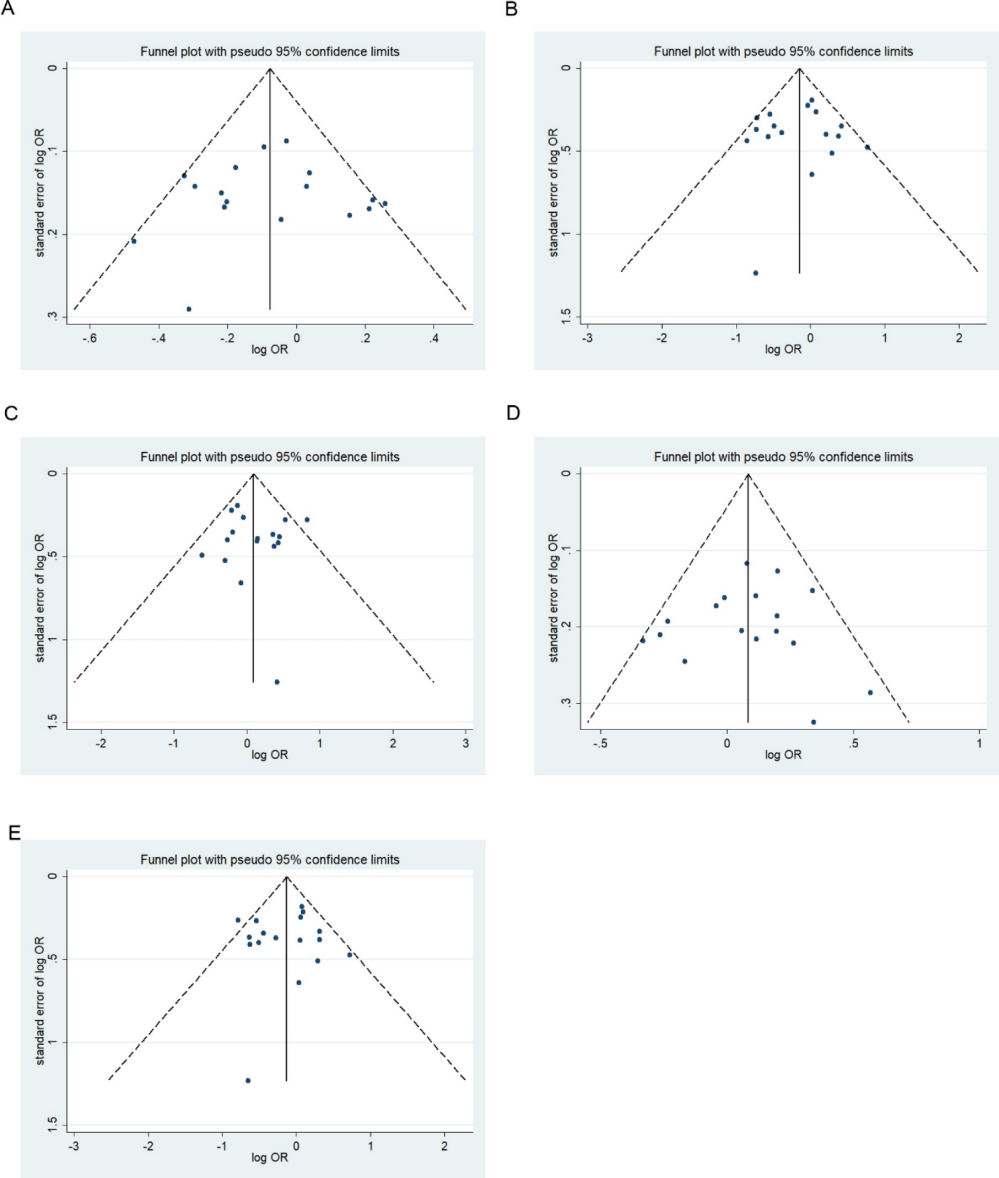
Funnel plot analysis depicting publication bias in the association between *MTHFR 1298A>C* polymorphism and schizophrenia in the five genetic models (A, allele contrast; B, homozygous codominant; C, heterozygous codominant; D, dominant; and E, recessive).

We assessed possible publication bias using a Begg’s funnel plot and Egger’s test. No obvious asymmetry was observed in the funnel plot and Begg’s test results, indicating a lack of publication bias (*p* > 0.05) except for the homozygous codominant model of 677C>T locus in BD (*p* = 0.025) (Figs 8–13).

## Discussion

The present meta-analysis included 66 studies that investigated the association between *MTHFR* (677C>T and 1298A>C) polymorphisms and occurrence of ADHD, BD and SCZ. Overall, our meta-analytical results provided evidence that *MTHFR* 677C>T was associated with occurrence of BD and SCZ, while the 1298A>C polymorphism was related to ADHD and BD. The sensitivity analysis indicated that these results were stable and reliable.

Five previous retrospective studies investigated the association between *MTHFR* polymorphisms and ADHD [2, 37–39, 77]. Our results were very similar to those of Tahereh Sadeghiyeh [77], but not exactly the same as those of Saliha Baykal and Emel Ergul [37, 38]. A total of five retrospective studies were included, which represented *MTHFR* polymorphisms more accurately than previous published studies. This is the first meta-analysis to include recent published studies concerning the association between *MTHFR* polymorphism and ADHD occurrence. Therefore, to some extent, our study provides a more reliable assessment of the association between *MTHFR* polymorphisms and ADHD. Additionally, some previous studies showed that ADHD occurrence was affected by various environmental factors [78]. It is possible that epigenetic risk mechanisms in ADHD responding to environmental risk factors or trans-regulatory and gene × environment effects in the development of child psychopathology might play a consequential role in ADHD etiology [79]. In addition, ADHD subtypes represent distinct clinical entities and may have different genetic backgrounds [80].

To date, case-control studies and meta-analyses have explored the role of *MTHFR* polymorphisms in BD occurrence [24, 31, 33, 43, 51, 81–83] but with no consistent conclusion. Additionally, The *MTHFR* gene polymorphism is unlikely to play a major role in the pathogenesis of obsessive-compulsive disorder [84]. Our study showed that the 677C>T and 1298A>C polymorphisms were involved in the occurrence of BD. Moreover, a genome-wide association study suggested that the *MTHFR* gene polymorphism was related to mood disorder [85]. The Genotypes of 677C>T were related to total homocysteine (tHcy), folate and B12. Individuals with TT genotype have elevated tHcy and reduced folate and B12 levels, which may be a susceptible factor for the BD [48]. The interaction of BDNF Val66Met and MTHFR C677T may reduce the hippocampal size in both healthy controls and patients with first-episode psychosis [86].

The C677T polymorphisms of *MTHFR* had an influence on SCZ symptoms. However, the effect of the T allele on the negative symptoms of SCZ could be further enhanced by folate deficiency [87]. Additionally, there was a significant association between the 677TT genotype and SCZ under the recessive model in the male patient subgroup, and CT genotype under the overdominant model in the total patient group [65]. The OR for patient with BD and SCZ in 1298CC homozygous state was 3.768 (P = 0.0003) and 2.694 (P = 0.0123), respectively. After the stratification of patients based on gender, only a significant association of 1298CC genotype with BD in female patients was observed (P = 0.0005) [46]. Moreover, a previous meta-analysis indicated that the T allele and TT genotype of C677T carriers showed significantly increased risk of major psychiatric disorders including SCZ and BD [33]. Moreover, the activity of MTHFR will be affected by multiple single-nucleotide polymorphisms. However, variations other than the 677C>T and 1298A>C polymorphisms have received little attention. In addition, aggravating symptoms, increased MTHFR polymorphisms, and reduced genomic methylation levels can be observed in patients with early-onset SCZ [88]. MTHFR 677T allele carriers have lower levels of total cholesterol and low-density lipoprotein cholesterol than those with the 677CC genotype [89]. There was a positive association between the COMT—MTHFR interaction and attention in inpatients suffering from recent onset SCZ [90]. MTHFR A1298C, but not C677T, was associated with the metabolic syndrome, its CC genotype having a 2.4 times higher risk compared to AA genotype [91]. In addition, the C allele of MTHFR was associated with BMI reduction in the schizophrenia patients following switching of antipsychotics to aripiprazole and ziprasidone [92].

There were several potential limitations to the present study. First, the most important was sample size. Small samples with limited participants are usually accompanied by selection biases. These studies lack sufficient power to support or refute meaningful conclusions [93]. Second, subgroup analysis cannot be carried out with limited samples, so the influence of some factors (e.g. ethnicity, source of controls and diagnostic criteria) were ignored. The discrepancies of the studies may result from population stratifications, explicitly, socio-economic status [94]. Finally, clinical subtypes of the mental disorder, gene–gene interaction and epigenetics were not examined in this study due to insufficient information.

## Conclusions

Our findings suggest that the *MTHFR* 677C>T was associated with occurrence of BD and SCZ, while the 1298A>C polymorphism was related to ADHD and BD. Studies involving larger sample sizes will be necessary to confirm the meta-analysis results, particularly in different ethnicities and to address the epigenetic mechanisms and environmental influences on the occurrence of common mental disorders.

## Supporting information

S1 Checklist Meta-analysis on genetic association studies checklist. (DOCX)
